# Characterisation of urinary WFDC12 in small nocturnal basal primates, mouse lemurs (*Microcebus spp.*)

**DOI:** 10.1038/srep42940

**Published:** 2017-02-22

**Authors:** Jennifer Unsworth, Grace M. Loxley, Amanda Davidson, Jane L. Hurst, Guadalupe Gómez-Baena, Nicholas I. Mundy, Robert J. Beynon, Elke Zimmermann, Ute Radespiel

**Affiliations:** 1 Centre for Proteome Research, Institute of Integrative Biology, University of Liverpool, Liverpool, L69 7ZB, UK; 2 Mammalian Behaviour & Evolution Group, Institute of Integrative Biology, University of Liverpool, Leahurst Campus, Neston, CH64 7TE, UK; 3 Department of Zoology, University of Cambridge, Downing Street, Cambridge, CB2 3EJ, UK; 4 Institute of Zoology, University of Veterinary Medicine, Hannover, Buenteweg 17, 30559 Hannover, Germany

## Abstract

Mouse lemurs are basal primates that rely on chemo- and acoustic signalling for social interactions in their dispersed social systems. We examined the urinary protein content of two mouse lemurs species, within and outside the breeding season, to assess candidates used in species discrimination, reproductive or competitive communication. Urine from *Microcebus murinus* and *Microcebus lehilahytsara* contain a predominant 10 kDa protein, expressed in both species by some, but not all, males during the breeding season, but at very low levels by females. Mass spectrometry of the intact proteins confirmed the protein mass and revealed a 30 Da mass difference between proteins from the two species. Tandem mass spectrometry after digestion with three proteases and sequencing *de novo* defined the complete protein sequence and located an Ala/Thr difference between the two species that explained the 30 Da mass difference. The protein (mature form: 87 amino acids) is an atypical member of the whey acidic protein family (WFDC12). Seasonal excretion of this protein, species difference and male-specific expression during the breeding season suggest that it may have a function in intra- and/or intersexual chemical signalling in the context of reproduction, and could be a cue for sexual selection and species recognition.

Chemical signalling is the evolutionarily oldest and most widespread communication mode among animals^1^. Chemical signals are highly variable, ranging from small volatile molecules to large non-volatile proteins. They are perceived by two olfactory systems; the main olfactory epithelium in the nasal cavity and the vomeronasal organ that is specialized in pheromone detection in mammals^2^. Chemical signals are typically deposited by specific glands or *via* urine or other body fluids and faeces. However, whereas there is a large body of literature on species-specific olfactory behaviours (e.g. scent marking), there is typically much less knowledge about the information content or biochemical composition of semiochemicals. A notable exception is provided by the domestic house mouse, the first mammal for which there is a comprehensive understanding of the role of low molecular weight volatile components, proteins and their interplay in semiochemistry. In the house mouse, proteins act both as pheromone binders and also as pheromones in their own right^3^,^4^,^5^,^6^. However, the extent to which such protein-mediated semiochemistry operates across the animal kingdom outside the myomorph rodents is unknown. The proteins of mouse urine, the major urinary proteins (MUPs), have multiple roles, including individual recognition, inter-male aggression, kin recognition and induction of learning^3^,^4^,^7^,^8^,^9^,^10^,^11^,^12^. MUPs are highly polymorphic^13^,^14^,^15^, eliciting the combinatorial complexity that is needed for such subtle semiochemical effects^15^,^16^,^17^,^18^ Hurst *et al*., in revision.

Mouse lemurs (*Microcebus spp*.) are small nocturnal solitary foragers constituting one primate genus endemic to Madagascar. They strongly rely on olfactory and acoustic signalling in adaptation to their dispersed nocturnal life style^19^,^20^. Scent marking behaviour of mouse lemurs is rich and includes urine washing, anogenital marking and substrate rubbing^20^,^21^. Mouse lemurs possess a functional vomeronasal organ (VNO^22^,^23^) and have a repertoire of over 200 vomeronasal receptor genes^24^,^25^ that are expressed in the VNO but also partially in the main olfactory epithelium^22^. Olfaction may be involved in various contexts of intraspecific communication, as in marking group ownership of sleeping sites^19^, or in many aspects of reproduction, and may be used within and between the sexes^20^,^26^,^27^,^28^,^29^.

Mouse lemurs reproduce strictly seasonally and produce one or two litters per year that are reared during the resource-rich rainy season^30^,^31^,^32^. Their mating system is a multi-male/multi-female system that involves male mouse lemurs competing intensely for access to oestrous females during the reproductive season^26^,^33^,^34^. On the other hand, females exert some level of mate choice^35^,^36^,^37^ while having to assure fertilization during the one or two very short receptive periods per year^28^. Physiological changes prior to the breeding season include decreased body mass (due to loss of adipose tissue) and increased testis size^38^,^39^. This testis size increase can be reversed subsequently in subordinate males by exposure to urine from a dominant male^40^.

A preliminary survey of *Mup* genes across non-rodent mammals proposed that two genes encoding MUPs could be located within the first draft of the grey mouse lemur (*Microcebus murinus*) genome^13^. However, there are no data on expression and tissue specificity for these putative proteins. In this study, we examined the urine of two species of mouse lemur *– Microcebus murinus* and *Microcebus lehilahytsara –* with the aim of identifying urinary proteins that have the potential to be involved in chemosignalling in a reproductive context or in species recognition. We focussed on two allopatrically-distributed species that are maintained under standardized conditions in the laboratory to identify potential species-specific differences. We report that mouse lemur urine does not contain detectable levels of MUPs, notwithstanding the two *Mup* gene paralogues reported by Logan and colleagues^13^. However, the urine of males from both species sometimes contains high levels of a low molecular weight, seasonally expressed protein. We gained sufficient protein-level information to permit identification of this protein and speculate on likely functions.

## Materials and Methods

### Animals

Urine samples were obtained from 24 (14 male, 10 female) *M. murinus* and from 13 (8 male, 5 female) *M. lehilahytsara* that belong to the captive mouse lemur colony at the Institute of Zoology, University of Veterinary Medicine Hannover. Samples were taken during the reproductive period (March–May 2011 or March–May 2012) and during the non-reproductive period (September 2011–January 2012). Housing conditions^41^,^42^ were in accordance with European Directive 2010/63/EU on the protection of animals used for scientific purposes, the German Animal Welfare Act, and corresponding section for animals used for scientific purposes and licensed by the Bezirksregierung Hannover (reference number: AZ 33.9-42502-05-10A080) and by the Ordnungsamt, Gewerbe- und Veterinärabteilung, Landeshauptstadt Hannover (AZ 42500/1H). Animals were housed in groups of two or three individuals of the same or opposite sex (depending on the breeding management decisions). The non-invasive procedure was approved by the animal welfare officer of the University of Veterinary Medicine Hannover, Foundation as well as by the State of Lower Saxony Office for Consumer Protection and Food Safety (LAVES; approval date: April 28, 2014; number: 33.12-42502-04-14/1454), which is the responsible agency of the State Lower Saxony for approval of animal studies according to the German Animal Welfare Act (TierSchG). The study is in accordance with the recommendations of the Weatherall report, “*The use of non-human primates in research*” (https://www.mrc.ac.uk/documents/pdf/the-use-of-non-human-primates-in-research).

The animals are kept in same or mixed-sex pairs or mixed-sex pairs with offspring. They are housed in cages measuring 150 × 62.5 × 80 cm for a single individual or 150 × 150 × 80 cm and 140 × 170 × 70 cm, respectively, for a pair. The diet consists of seasonal fresh fruits and vegetables, dried fruits, and mealworms every other day. On non-mealworm days, the animals are offered milk porridge enriched with vitamins, minerals, and albumin^41^. Water is available *ad libitum*. Environmental enrichment is provided within the housing cages, branches and hollow cylinders allow the animals to climb and hide. To simulate the natural condition, where mouse lemurs sleep, rest and rear their offspring in tree holes^43^, each cage is equipped with several sleeping boxes (20 × 11 × 11 cm each) that are also occasionally used to collect urine samples. Urine sampling also took place opportunistically by pipetting during the weekly handling routine handling when each individual is constrained for one to two minutes in hand when body mass, health condition and reproductive status are assessed. Suffering can be stated to be minimal, since the animals are used to these weekly routines.

### Urine collection

Samples were obtained by direct sampling during weekly handling routines or with a urine collection box. During the handling routines, the urine was collected with a disposable 1 mL pipette whenever the animals urinated spontaneously. Alternatively, the animals were confined to a urine collection box (20.5 × 12 × 13 cm, length × depth × height) for 30–90 min at the beginning of their activity period. The urine passed through a mesh at the bottom of the box and was collected by a stainless steel funnel. The collection box and funnel were cleaned thoroughly after every use. All urine samples were frozen at −20 °C within 2 h of collection. For some lemurs, multiple samples were collected (see Supplementary Data) but have been treated as independent measures.

### Protein characterization

Total protein concentration was measured using a Coomassie Plus protein assay kit (Pierce, Rockford, USA) using bovine serum albumin as standard. SDS-PAGE was conducted using standard protocols. Creatinine concentration was measured by the alkaline picrate assay kit from Sigma-Aldrich.

### Electrospray ionisation mass spectrometry

Urine samples were diluted in formic acid (0.1% (v/v) in HPLC grade water) to a protein concentration of approximately 5 pmol/μl. The samples were injected onto a C4 desalting trap (Waters MassPREP™ Micro desalting column, 2.1 × 5 mm, 20 μm particle size, 1000 Å pore size) (Waters, Manchester, UK) that was fitted on a Waters nano ACQUITY Ultra Performance liquid chromatography^®^ (UPLC^®^) system. The chromatography system was coupled to a Waters SYNAPT™ G1 QTof mass spectrometer fitted with an electrospray source. Protein was eluted over a 10 min acetonitrile (ACN) gradient (5–95% (v/v)) at 40 μl/min. Data were collected between 500–3500 m/z. The data were processed using maximum entropy deconvolution (MAX ENT 1, Mass Lynx version 4.1, Waters) at 0.5 Da/channel over a mass range of 8500–10000 Da. The mass spectrometer was calibrated externally with horse heart myoglobin (1 pmol/μl, Sigma).

### In-gel digestion

Pieces of SDS-PAGE gel containing protein bands were washed repeatedly with 25 mM NH_4_HCO_3_, ACN (50:50) for 15 min at 37 °C until the gel pieces were fully destained. The gel plugs were then reduced in dithiothreitol (10 mM) at 60 °C for 1 h. The dithiothreitol solution was discarded and cysteine residues were alkylated with iodoacetamide (25 μl, 55 mM) in the dark at room temperature for 45 min. The recovered gel pieces were dehydrated in ACN for 15 min at 37 °C. Proteolytic enzymes – trypsin, endoproteinase LysC or endoproteinase GluC (each 10 μl, 10 ng/ml) were added to each of the gel pieces and incubated for 16 h. The digestion was stopped by the addition of formic acid (final concentration 1% v/v).

### MALDI-TOF analysis

The peptide mixtures from in-gel digestion were analysed by MALDI-TOF-MS on a Bruker ultrafleXtreme™ mass spectrometer in reflectron mode. Samples were mixed with MALDI matrix (saturated solution of α-cyano-4-hydroxycinnamic acid in 50% (v/v) ACN/0.2%(v/v) trifluoroacetic acid (TFA)) in a 1:1 ratio and spotted onto a target plate before being left to air dry. The laser frequency was 1000 Hz, laser energy 27% of maximum and 500 laser shots were collected per spectrum, between 800–4000 m/z.

### In solution digestion

Samples, diluted in 25 mM NH_4_HCO_3_ to 10 μg protein in a 50 μl digest were incubated with RapiGest™ SF Surfactant (0.05% w/v final concentration, Waters, Manchester, UK) at 80 °C for 10 min. The samples were then reduced with dithiothreitol (3 mM final concentration) at 60 °C for 10 min followed by alkylation with iodoacetamide (9 mM final concentration) in the dark at room temperature for 30 min. The protease, either trypsin (0.2 μg/μl diluted in 25 mM NH_4_HCO_3_) or endoproteinase LysC (0.1 μg/μl diluted in 25 mM Tris HCl pH 8.5), was added to the digests at a substrate:enzyme ratio of 50:1 and left to incubate for 16 hours. Following incubation, a small portion of the digested material was removed to run on an SDS-PAGE gel to check for complete digestion. The rest of the digest was treated with TFA (to a final concentration of 0.5% v/v) and incubated at 37 °C for 45 min to precipitate the RapiGest™ SF Surfactant prior to LC-MS analysis. The samples were then centrifuged at 10,000 rpm for 15 min and the supernatant transferred to a fresh 0.5 ml Eppendorf.

### LC-MS analysis

Digests were analysed using an Ultimate 3000 nano system (Dionex/Thermo Fisher Scientific, Hemel Hempstead, UK) coupled to a QExactive mass spectrometer (Thermo Fisher Scientific, Hemel Hempstead, UK). Peptides were loaded onto a trap column (Acclaim PepMap 100, 2 cm × 75 m inner diameter, C18, 3 μm particle size, 100 Å pore) at 5 μL/min with an aqueous solution containing 0.1%(v/v) TFA and 2%(v/v) ACN. After 3 min, the trap column was set in-line with an analytical column (Easy-Spray PepMap^®^ RSLC 15 cm × 75 μm inner diameter, C18, 2 μm, 100 Å) (Dionex/Thermo Fisher). Peptides were eluted by using an appropriate mixture of solvents A and B. Solvent A was 0.1%(v/v) formic acid in HPLC-grade water, and solvent B was 0.1% (v/v) formic acid in HPLC grade acetonitrile 80% (v/v). Separations were performed by applying a linear gradient of 3.8% to 50% solvent B over 35 min at 300 nL/min followed by a washing step (5 min at 99% solvent B) and an equilibration step (15 min at 3.8% solvent B). The mass spectrometer was operated in data dependent positive (ESI+) mode to automatically switch between full scan MS and MS/MS acquisition. Survey full scan MS spectra (300–2000 m/z) were acquired in the Orbitrap at 70,000 resolution (200 m/z) after accumulation of ions to 1 × 10^6^ target value based on predictive automatic gain control (AGC) values from the previous full scan. Dynamic exclusion was set to 20 s. The 10 most intense multiply charged ions (z ≥ 2) were sequentially isolated and fragmented in the octopole collision cell by high energy collisional dissociation (HCD) with a fixed injection time of 120 ms and detected in the Orbitrap at 35,000 resolution. The mass spectrometer conditions were as follows: spray voltage, 1.9 kV, no sheath or auxillary gas flow; heated capillary temperature, 250 °C; normalised HCD collision energy 30%. The MS/MS ion selection threshold was set to 1 × 10^4^ counts and a 2 m/z isolation width was set. Peak lists were generated using Proteome Discoverer (Thermo Fisher) and MASCOT (Matrix Science, London) as search engine. The search parameters were set to accept 1 missed cleavage, a fixed modification of carbamidomethylation of cysteine and variable methionine oxidation. Precursor and fragment ion error tolerances were set to 10 ppm and 0.01 Da respectively. Fragmentation was set to HCD. The MS/MS data were first searched against a database of all mammalian protein sequences in the SwissProt database, prior to searching all primate protein sequences from UniProt. Once the sequence of the protein was determined, a database entry was created containing the protein sequence from both mouse lemur species to assist in peptide coverage analysis.

### Data analysis

Urinary protein output (μg protein/μg creatinine) was log transformed to meet assumptions of parametric analysis (Shapiro-Wilks, P > 0.05). To take into account the variable number of samples available from different individuals, we used R (version 3.2.5) and the *lme4* test package^44^ to examine the effects of donor sex and season on protein output in a linear mixed effects analysis, with individual donor and species included as random effects (insufficient breeding season samples were available from *M. lehilahytsara* to include species as a main effect in analysis). As there was a nearly significant interaction between donor sex and breeding season, separate models examined the effect of breeding season on protein output within each sex. Visual inspection of residual plots did not reveal any obvious deviations from homoscedasticity and the distribution of residuals from each model approximated normality (Shapiro-Wilks, P > 0.05). Likelihood ratio tests compared the full model against a reduced model without the effect of interest using the anova function.

Sequencing analysis *de novo* of the mouse lemur protein was assisted by PEAKS software^45^ for proteomics (Bioinformatics Solutions Inc, Canada). Precursor and fragment ion error tolerances were set to 10 ppm and 0.01 Da respectively. Post translational modifications, carbamidomethylation (fixed modification) and oxidation of methionine (variable modification) residues were also included in the processing set-up. Fragmentation type was set to higher-energy C-trap dissociation (HCD). The average local confidence score – a score assigned by PEAKS which reflects the likelihood of a peptide sequence being correct – was set to a 55% cut off as recommended by the software vendor. The *de novo* sequenced peptides were then analysed using BLAST, (http://blast.ncbi.nlm.nih.gov), and compared to annotated exon/intron structure of *WFDC12* entries in Ensembl. The complete mature protein sequence was used to direct homology modelling using the Phyre2 server^46^. To confirm the sequence, the LC-MS/MS data was then searched against the newly constructed protein sequence in PEAKS, using database search tools PEAKS DB, which matched the *de novo* sequences generated against a database of all primate protein sequences in UniProt with the addition of the constructed protein sequence, and SPIDER, to search for mutations and sequencing errors. Search parameters included precursor and fragment ion error tolerances of 10 ppm and 0.01 Da, respectively. For the PEAKS DB search, a fixed modification of carbamidomethylation was set, and a maximum of 3 variable modifications (restricted to oxidation of methionine) per peptide was allowed. No non-specific cleavage was allowed, and the maximum number of missed cleavages was set to 2. The same error tolerances were used for the PEAKS PTM search^47^, which matched additional peptides from the *de novo* sequences to the database by searching for mass changes corresponding to a default list of 484 variable modifications, and the SPIDER search^48^, which attempts to find mutations and sequencing errors based on combining the information gained from the *de novo* sequences generated and the database sequence. For both searches, a threshold peptide confidence score (−10 logP) of 30 was used. PEAKS PTM also maintained the cleavage parameters set from the PEAKS DB search, whereas SPIDER automatically searched for non-specific cleavages. A false discovery rate of 1% was used for each search tool, and an ALC (average local confidence) cutoff score of 50% was used for *de novo*-only peptides. The mass spectrometry proteomics data have been deposited to the ProteomeXchange Consortium via the PRIDE^49^ partner repository with the dataset identifier PXD005196 and 10.6019/PXD005196.

Protein modelling was conducted using the Phyre2 server (http://www.sbg.bio.ic.ac.uk/~phyre2) in intensive mode^46^.

## Results and Discussion

**Figure 1:**
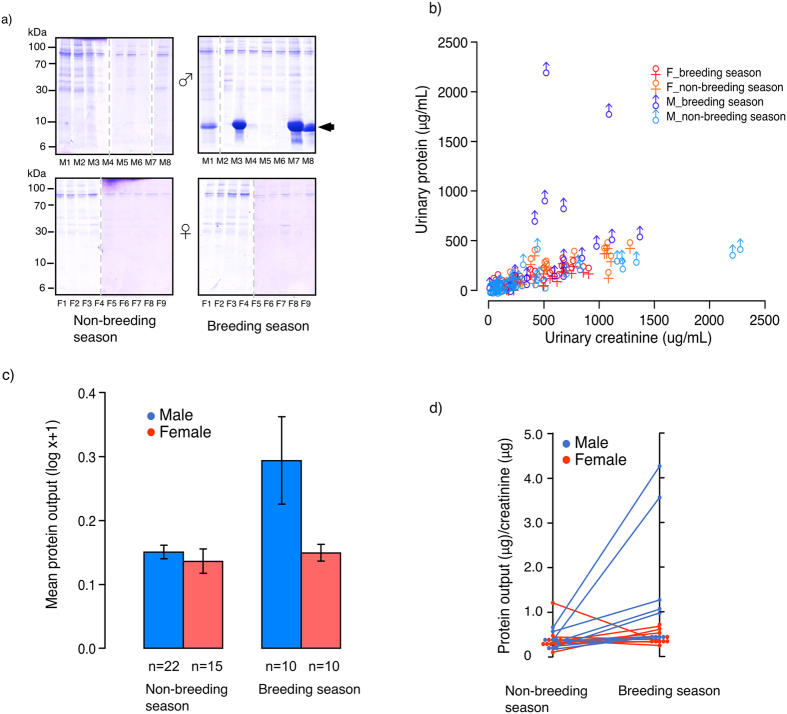
Protein content of mouse lemur urine. A group of 116 male and female mouse lemur (*M. murinus* and *M. lehilahytsara*) urine samples were analysed by SDS-PAGE (panel a, samples from several individuals, males: M1 to M8, females F1 to F9). Total urinary protein concentration generally increased with urinary creatinine, which provides a measure of urine dilution (panel b, symbols and colours represent donor sex and season). Some males in the breeding season had much higher levels of urinary protein than explained by urine dilution. The urinary protein output (expressed as μg protein/μg creatinine, averaged over replicate samples from the same individual and season) was higher in the breeding than in the non-breeding season among males, while there was no seasonal effect on female output (panel c, data are means ± sem, n indicates total number of individuals sampled). Multiple samples obtained from some individuals (10 males, 10 females) allowed urinary protein output to be compared between breeding and non-breeding season within the same individual (panel d).

Urine samples (116 samples in total, from 37 individuals) were collected from both male and female mouse lemurs (*M. murinus and M. lehilahytsara* data are combined in Fig. 1) during the breeding and the non-breeding season (details of samples are in Supplementary Table S1). The urine was initially analysed by 1D SDS-PAGE (Fig. 1a). No band corresponding to a MUP-like protein at 19 kDa was observed, but there was a predominant protein band at approximately 10 kDa present in some male samples from either species, exclusively during the reproductive season. No predominant protein was identified in female mouse lemur urine from either species, whether in or outside of the reproductive season (Fig. 1a). The concentration of protein varied between 0.08 mg/ml and 2.2 mg/ml across all animals and samples. However, there was a strong relationship between protein and creatinine concentration (r^2^ = 0.66, [protein] = 0.26 * [creatinine] + 40.6; Fig. 1b), most likely reflecting differences in urine dilution between samples. To correct for urine dilution, protein output was expressed as μg protein/μg creatinine. The effects of donor sex and breeding season on urinary protein output (data log transformed to meet assumptions for parametric analysis) were assessed using linear mixed effects models, with individual donor and species included as random effects. Confirming our initial observations from SDS-PAGE bands, seasonal effects on protein output appeared to vary between the sexes (interaction between breeding season and sex: ^2^ = 3.00, 1df, P = 0.08; Fig. 1c). Separate models were thus run to assess seasonal variation in urinary protein output within each sex. Among males, protein output was significantly elevated during the breeding season compared to the non-breeding season, a difference that was highly significant (effect of season: ^2^ = 12.99, 1df, P = 0.0003, Fig. 1c). In the breeding season, male output ranged from 0.13 to 5.46 mg protein/mg creatinine (mean ± SEM: 1.19 ± 0.34, n = 18 samples from 10 individuals), compared to a non-breeding season range of 0.10 to 1.12 (mean ± SEM: 0.43 ± 0.04, n = 50 samples from 22 individuals). By contrast, protein output showed no seasonal variation among females (effect of season: ^2^ = 1.12, 1df, P = 0.29, Fig. 1c) and was very similar to the output of non-breeding males (female mean ± SEM: 0.41 ± 0.05, n = 48 samples from 15 individuals, range 0.09 to 1.76). Among ten males that were sampled during both the breeding and non-breeding season, eight had higher urinary protein output in breeding season samples (Fig. 1b,d). Breeding season samples from these males revealed a strong 10 kDa band on SDS-PAGE. Of eight breeding season *M. murinus* males, three had exceptionally high levels of protein in urine, as did both of the breeding season *M. lehilahytsara* males that were sampled.

**Figure 2:**
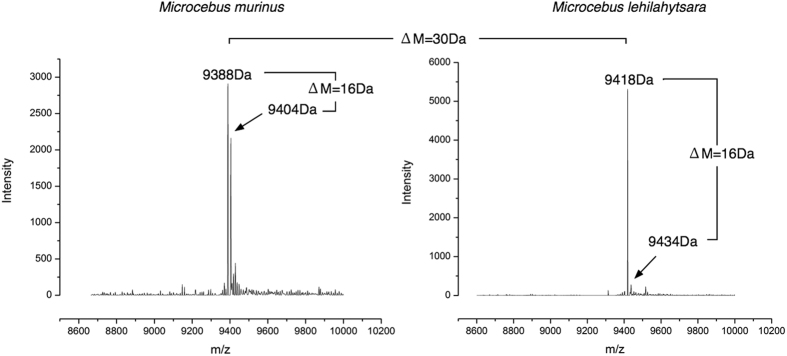
Intact mass analysis of the mouse lemur urinary protein. For urine samples from specific male mouse lemurs within the breeding season, the protein concentration was sufficiently high for analysis of the intact mass of the predominant protein by electrospray ionisation mass spectrometry. Urine samples were analysed for the two mouse lemur species, *M. murinus* and *M. lehilahytsara*, and representative deconvoluted mass spectra, showing the intact mass profiles are presented.

We have previously used mass spectrometry-based intact mass analysis to successfully characterise predominant urinary proteins in other species, and to ascertain the extent of any mass-resolvable heterogeneity^50^,^51^,^52^. Male mouse lemur urinary proteins were therefore analysed by electrospray ionisation mass spectrometry of diluted and desalted urine (Fig. 2). A protein of molecular mass 9,388 Da was identified in all male *M. murinus* samples that also demonstrated a strong band at approx. 10 kDa on SDS-PAGE – in turn these were all from males in the breeding season. A second mass peak, 16 Da heavier, was also identified; we surmised that this was probably due to partial oxidation of the main protein peak. Analysis of the urinary proteins in *M. lehilahytsara* urine similarly revealed a dominant protein of molecular mass 9,418 Da (30 Da heavier than the protein from *M. murinus*) in the male-derived samples that also expressed a 10 kDa SDS-PAGE band. These too were all from breeding season males. This protein mass was also partnered by a second mass peak, 16 Da heavier, as observed with *M. murinus.* In both species, the mass profile was simple, exhibiting none of the heterogeneity that we have previously observed for the MUPs of the domestic mouse^11^,^16^,^52^.

**Figure 3:**
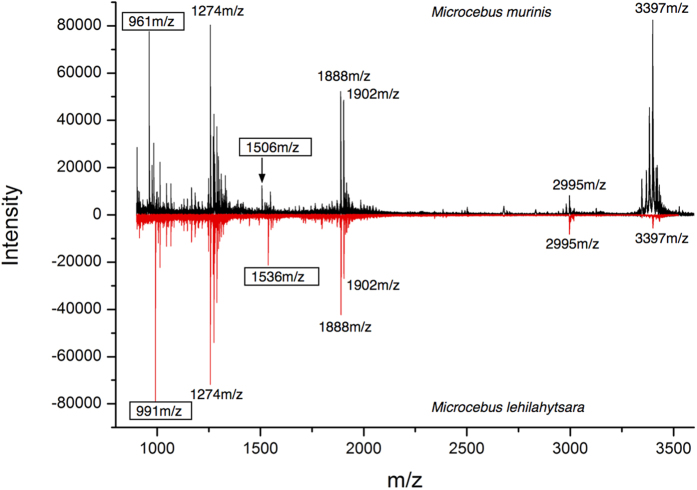
MALDI-TOF mass spectrometry of the mouse lemur urinary protein. For some breeding season males, the predominant 10 kDa band was resolved by SDS-PAGE and the gel fragment was processed by reduction, carbamidomethylation and digestion with trypsin. The resultant peptides were analysed by MALDI-TOF mass spectrometry. Representative spectra are included for *M. murinus* (plotted in a positive direction, black) and *M. lehilahytsara* (plotted in a negative direction for spectral comparison, red). The two boxed peptides exhibited a 30 Da mass difference between the two species.

To investigate the similarity of the 10 kDa protein in the two species, urine samples from breeding season males were resolved by SDS-PAGE, proteolysed by in-gel digestion and the tryptic peptides were analysed by MALDI-TOF mass spectrometry (Fig. 3). Many of the peptides were of the same mass in the digest from either species, very good evidence for the proteins being orthologues. However, two peptides (singly charged ions, [M + H]^+^, at m/z 991 and m/z 1536) in the peptide mass fingerprints from *M. lehilahytsara* samples were 30 Da heavier than cognate peptides in *M. murinus* derived samples ([M + H]^+^ m/z 961 and m/z 1506). The measured mass difference between the proteins from different species (30 Da, Fig. 2) was thus reflected in two tryptic peptides, most likely explained by localisation of amino acid change(s) to one fully digested tryptic peptide and a second peptide resulting from a tryptic miscleavage. A third peptide (m/z 2979 in both species) was accompanied by a +16 Da equivalent (m/z 2995) in both species; this probably contained the putative site of oxidation.

**Figure 4:**
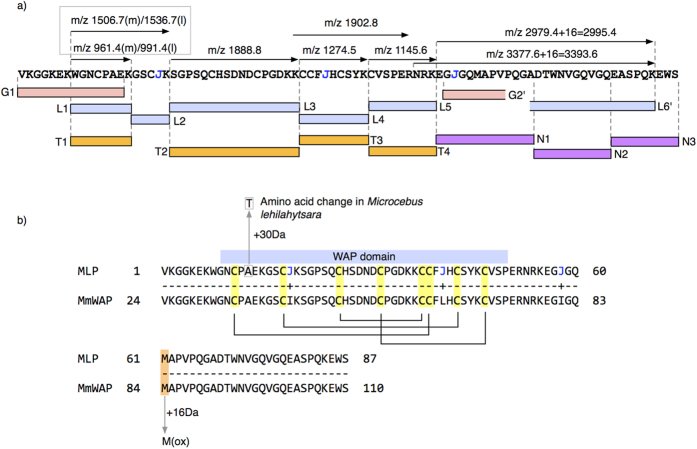
Complete sequencing strategy for the predominant 10 kDa protein. Urine from *M. murinus* and *M. lehilahytsara*, containing high levels of the 10 kDa protein was reduced, carbamidomethylated and digested in solution with four different endoproteolytic enzymes (G: endopeptidase GluC, L: endopeptidase LysC, N:endopeptidase AspN and T: trypsin), the peptides were separated by reversed phase chromatography and sequenced *de novo* by high resolution tandem mass spectrometry. The top panel (**a**) summarises the sequencing strategy (the symbol ‘J’ is used to define ambiguity between isobaric leucine and isoleucine) The bottom panel (**b**) indicates the homology (MLP: mouse lemur protein sequenced in this study) with the protein inferred from the draft genome sequence for *M. murinus*, (MmWAP, number starting from 24 to accommodate the predicted signal peptide 1–23) assuming the alternate splice variant (see Fig. 5). The putative disulphide bond arrangement observed for other proteins containing the four disulphide core is superimposed on the new protein sequence. Additionally, the position of the single amino change between *M. murinus* and *M. lehilahytsara* and the site of methionine oxidation (for both species) are indicated. The masses of peptides observed by MALDI-TOF mass spectrometry are highlighted at the top of panel a, and the boxed peptide masses confirm the observations from MALDI-TOF mass spectra, where the observed peptides for *M. murinus* (m) or *M. lehilahytsara* (l) are identified.

To better characterise the 10 kDa protein, we completed a full protein sequence analysis by mass spectrometry based sequencing *de novo*. The proteins in urine samples containing elevated levels of the 10 kDa from both species were digested with four different proteases – trypsin, endopepeptidase LysC, endopeptidase GluC and endopeptidase AspN to generate peptides that would thus overlap and permit reconstruction of the primary sequence, with the single caveat that the isobaric Leu/Ile amino acid pair could not be discriminated by this method. The protein digests were analysed by LC-MS/MS and the peptide fragmentation spectra were interpreted to yield *de novo* protein sequence data using PEAKS7 software. Initially, high-scoring individual peptide sequences were searched against protein databases using BLAST (http://blast.ncbi.nlm.nih.gov). Several peptide sequences partially matched to a Whey Acidic Protein (WAP) identified in the ring-tailed lemur, *Lemur catta* (Uniprot accession A4K2S4). This protein, full name ‘WAP four – disulphide core domain 12 (WFDC12)’, was therefore also used to assist the assembly of overlapping peptides (obtained by using endopeptidases of different specificities) for which sequence data was obtained, as a result of which we were able to reconstruct an 87 amino acid sequence (Fig. 4). Sequence analysis of the *M. lehilahytsara* protein also revealed an 87 amino acid protein that was almost identical to the protein from *M. murinus*. However, there was clear evidence for a single amino substitution in one peptide; corresponding to the singly charged m/z 961 in *M. murinus* and cognate peptide at m/z 991 in *M. lehilahytsara* observed in MALDI-TOF (Fig. 3). The m/z 961 (*M. murinus*) peptide was sequenced as WGNCP**A**EK, whereas the m/z 991 peptide (*M. lehilahytsara*) was sequenced as WGNCP**T**EK. This Ala- > Thr substitution accounted for the 30 Da difference in mass. Further, the miscleaved peptide WGNCPAEKGCSIK from *M murinus* would explain the MALDI-TOF peptide at [M + H]^+^ 1506 m/z that was present as a 30 Da heavier peptide in *M lehilahytsara* (Fig. 3). The protein peak that was evident as 16 Da heavier on the intact mass spectrum in both species was attributable to oxidation of the single methionine residue in the m/z 2979 tryptic peptide (ELGQG**M**APVPQKDTWNVGQVGQEASPQK) leading to a peptide at m/z 2995 (ELGQGM*APVPQKDTWNVGQVGQEASPQK) where * indicates the site of oxidation. This is the only methionyl residue in the protein sequence. Finally, the MALDI-TOF peptide at m/z 1902 is most readily explained as the sequence KCCFLHCSYKCVSPER (predicted mass 1901.8 Da) that contained four cysteine residues. It is possible that this peptide had evaded reduction and alkylation – the tight disulphide bond ‘knot’ (see later) could preclude full access to these reagents.

The predicted mass of the newly determined primary protein sequence from *M. murinus* was 9,396 Da, 8 Da higher than the 9,388 Da major peak observed by electrospray ionisation mass spectrometry (Fig. 2). However, a highly conserved feature of WAP domain proteins is the presence of eight cysteinyl residues that form four disulphide bonds^53^,^54^,^55^. The oxidation of eight cysteinyl residues to four disulphide bonds would result in an 8 Da mass decrease, resulting in a predicted mass of 9,388 Da, exactly concordant with the observed mass. Identical arguments for the protein from *M. lehilahytsara* meant that the observed intact mass was also accurately predicted by the protein sequence obtained by sequencing *de novo*, including the single conservative amino acid change (Ala to Thr).

**Figure 5:**
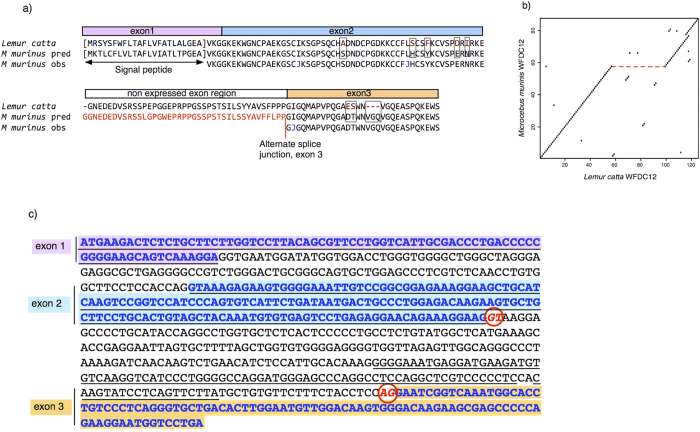
Sequence comparison between mouse lemur and ring tailed lemur. Panel (a) the predicted and observed mouse lemur sequences were aligned with the sequence from the ring tailed lemur (*Lemur catta*, Uniprot accession number A4K2S4). The position of an alternate splice junction in the mouse lemur sequence (http://www.ncbi.nlm.nih.gov/protein/829731644/) is indicated in red. The ambiguity between the isobaric pair leucine and isoleucine is indicated by the letter ‘J’. The dot plot (panel b) comparing the mouse lemur and ‘long form’ predicted ring tailed lemur sequences was generated using the dotPlot routine within the *seqinr* R package (http://seqinr.r-forge.r-project.org/) with a window size of five and scoring matches of three or more amino acids within the window. Panel (c) - Inferred exon/intron structure of *WFDC12* in *Microcebus murinus* genomic sequence of *WFDC12* from Ensembl record ENSMICG00000000986. The four exons from automated prediction are underlined. The three inferred actual exons that encode the WFDC12 protein sequenced in this study are in blue font with coloured background – the translation of these 3 spliced exons is shown as “*M murinus* pred” in panel b. Exons 1 and 2 and intron 1 are the same in both annotations. The predicted 5′ and 3′ splice sites for the newly defined intron 2 are circled and coloured red, with an AG 3′ splice site immediately upstream of exon 3, as expected.

The predicted mass of the protein sequence from *Lemur catta* (Uniprot accession A4K2S4) used to aid assembly of the peptides was 13.2 kDa, significantly larger than the intact mass observed for the mouse lemurs (9.4 kDa). Moreover, there was a region of the *L. catta* protein sequence that was not mapped by any of the peptide *de novo* sequencing data. Failure to acquire evidence for this region of the protein sequence could be for one of two reasons; first, that this region was missing in the mouse lemur sequence or secondly, that this region of the protein has evolved so significantly that homology matching was no longer achievable. The lower mass of the mouse lemur protein favoured the first explanation. When the entire mouse lemur sequence obtained by sequencing *de novo* was used in a BLAST protein search, the strongest match was to a genomic sequence encoding a putative 148 amino acid WAP four-disulfide core domain protein 12 (WFDC12) from *L. catta*. The mouse lemur protein aligned well (75/128 identities, 59%), and all cysteine residues aligned perfectly. However, no sequences aligned to the middle section of the *L. catta* protein (coloured red in Fig. 5a). As the mouse lemur protein was only 9.4 kDa compared to 13.2 kDa for the mature *L. catta* protein, this is corroborative evidence that the corresponding middle section of the sequence of the *M. murinus* protein was absent. Certainly, the overall similarity between the *L. catta* and the *M. murinus* proteins is evident from the dot plot analysis of the two sequences (Fig. 5b). Using the protein sequence described here to search the draft mouse lemur genome (version 1.0) also revealed a match to a putative protein coding gene that also included a nucleotide sequence encoding the ‘missing’ portion of the grey mouse lemur protein that has been proposed for *L. catta* (Fig. 5a).

Thus, the predicted sequences from the mouse lemur (and the paralogue from the ring-tailed lemur) genome data were at variance with the observed intact mass analyses from the two species of mouse lemur. Closer examination of the *M. murinus* sequence revealed the potential for an alternative intron/exon structure that would eliminate the coding region for the peptide tract that was not observed (Fig. 5c). The splicing alternatives could thus generate a ‘long form’ and a ‘short form’ of the mouse lemur protein. All evidence obtained from urinalysis is consistent with expression and secretion of the short form. Indeed, peptide mapping from multiple individuals to the long form of the protein was compellingly in favour of the short form - in no individual was there evidence for peptides that would be manifest by the long form (Supplementary Fig. S1). Thus, we provide unambiguous evidence for expression of this gene and release of the gene product in male *M. murinus* urine in the breeding season. Evidence of expression of the same ‘short form’ expression was paralleled in *M. lehilahytsara*. A different prediction algorithm (Gnomon; http://www.ncbi.nlm.nih.gov/genome/guide/gnomon.shtml) did however predict the ‘short form’. Despite the high level expression in some breeding season males, the protein sequenced herein matched exactly a predicted protein encoded by a mouse lemur cDNA (accession number XP_012635896; http://www.ncbi.nlm.nih.gov/protein/829731644/). This cDNA was derived from female kidney. Expression in kidney (albeit female) could be consistent with the appearance of this protein in urine. However, it is not possible to infer the site of production of the protein in males specifically within the breeding season.

Confirmatory sequence matching was obtained from in-solution digestion of urinary proteins from multiple individuals. Alignment of all the peptides that were analysed by multiple LC-MS runs provided strong evidence for short form peptides, whereas no peptides corresponding to the additional sequence in the long form were detected (Supplementary Fig. S1). Our data confirm that in urine at least, the ‘short form’ is expressed, with little evidence for the ’long form’. However, the initial evidence of a ‘long form’ of this protein derived from the genomic sequence of *L. catta*. To explore this ambiguity further, we were able to acquire urine samples from the communal latrine of 5 male *L. catta* housed at Erlebnis Zoo, Hannover that were collected three times a day, once a week, for three weeks during December and 6 weeks during April-June. We performed creatinine-normalised LC-MS/MS analysis on the tryptic peptides from nine urine samples, matching observed peptides to the predicted long form protein in this species. We additionally performed Glu-C digests on the two samples with the highest abundance of WFDC12, to allow for the large size of a predicted tryptic peptide in the ‘long’ form, less likely to be seen by tryptic digest. All data was again analysed by PEAKS. As with *M. murinus* and *M. lehilahytsara*, there was no evidence for the long form in this species. We conclude that the short form of the WFDC12 protein is also expressed in *L. catta* urine (Supplementary Fig. S2). As with *M. murinus*, the possibility of an alternate splice site is evident in the *L. catta* sequence (Supplementary Fig. S3).

The mouse lemur urinary protein is a member of the ‘whey acidic protein four disulphide core’ (WFDC) family, specifically WFDC12. This group of proteins, containing eight characteristically spaced cysteinyl residues that form four disulphide bonds, share limited overall sequence identity, except for the conserved cysteine-rich region and the precise positioning of the cysteine residues^53^. Unlike many other WFDC proteins, WFDC12 is restricted to a short N-terminal segment, the four disulphide core and a C-terminal amino acid section (approx. 40 amino acids) of protein sequence that has no recognised domain structure. BLAST searching of the C-terminal domain *from M. murinus* only returned matches to the *Microcebus murinus* (accession: XP_012635896) and the *L. catta* (accession: A4K2S4) WFDC12 sequences, (the other lemur sequence present in the protein database, from Coquerel’s sifaka (accession XP_012513356), did not elicit a match). Indeed, the C-terminal sequence from the sifaka more closely resembled that of other primates. This C-terminal sequence may therefore be specific to the mouse lemurs, a notion that must await further molecular data from other lemur species.

**Figure 6:**
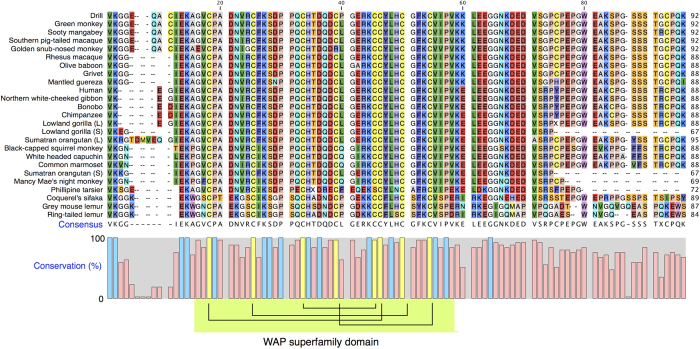
The primate WFDC12 family. All available primate WFDC12 proteins, were aligned using the CLC Sequence Viewer (www.clcbio.com), version 7.6.1. The eight fully conserved cysteine residues are coloured yellow in the conservation graph, in addition to the further 15 residues (blue) within the four disulphide domain that are conserved across all primates shown here.

Inferred WFDC12 protein sequences from multiple primates can be readily aligned with the mouse lemur protein (Fig. 6). The position of the eight cysteine residues in the mouse lemur proteins match almost perfectly the arrangement in other primates, confirming the role of the core fold in these proteins (we note that in two sequences, one of the eight cysteine residues has been replaced, but it is not clear if these reflect sequencing uncertainty). In addition, a further 15 residues are fully conserved across all primates from the mature protein N-terminus to 10 residues C-terminal to the last cysteine residue of the four disulphide core. After approximately 10 residues C-terminal to the four disulphide core domain, the sequences diverge very noticeably. For four primates, *Gorilla gorilla* (gorilla; a long and short form are listed in the genomic database), *Pongo abelii* (Sumatran orangutan, also a long and short form), *Aotus nancymaae* (Nancy Ma’s night monkey) and *Carlito syrichta* (Philippine tarsier) the protein sequences are predicted to terminate between 10 and 20 amino acid residues from the core. It is possible that these truncated forms reflect uncertainties in early drafts of genome sequences. For the remaining primates a common feature is a C-terminal extension approx. 50 amino acids long.

**Figure 7:**
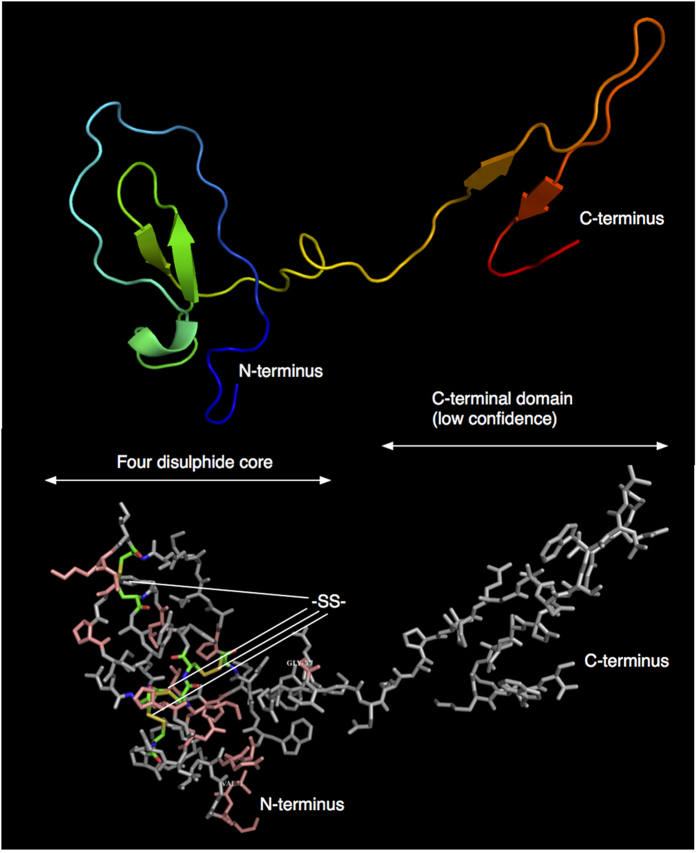
Homology modelling of the mouse lemur urinary protein. The sequence of the mouse lemur protein from *M. murinus* was used to obtain a model structure using the Phyre2 server (http://www.sbg.bio.ic.ac.uk/~phyre2) using the ‘intensive’ modelling approach. The four disulphide core was modelled to a very level of confidence, but the C-terminal domain cannot be predicted with equivalent confidence.

Within primates, a total of 25 residues at the N-terminal region of the protein, containing the four disulphide core, were completely conserved, suggesting conservation of structure. To gain further insight into the mouse lemur protein, we conducted homology modelling using the Phyre2 package^46^. The disulphide linked core was modelled with confidence (scores > 99), using the known three dimensional structures of other proteins that contain this WFDC domain, including antileukoprotease (2Z7F.PDB) and elafin-like knottins (1FLE.PDB, 2REL.PDB, 1UDK.PDB). The four disulphide core was confidently modelled, and the positions of the eight cysteine residues were optimally disposed to generate the four disulphide bonds (Fig. 7). The 15 residues that are conserved in all primate sequence known to date are disposed predominantly towards the exterior of the core fold. The C-terminal domain could not be modelled confidently as there was no specific template against which a structure could be modelled, but it is noteworthy that there is potential for a flexible region between the N- and C-terminal regions.

At present, it is not feasible to make confident assertions about the function of this protein. The high level of expression in males, restricted to the breeding season, is consistent with a role in reproduction or in social interaction during the breeding season. Moreover, not all breeding season males expressed this protein, suggesting that it would be worth exploring a relationship between expression and social hierarchy. Biological functions of some of the WFDC proteins include protease inhibition and antibacterial/antimicrobial activity^56^. The inhibitory loop in WFDC protease inhibitors is not, however, conserved in WFDC12, and preliminary experiments have shown that the mouse lemur WFDC12 is not capable of acting as an inhibitor of pancreatic trypsin or pancreatic elastase (Unsworth, unpublished data). Although the function of this protein in primates is currently unknown, the orthologue of WFDC12 in the mouse (also known as Swam2) has antibacterial activity^56^. Putative antimicrobial activity requires further analysis.

The production of large molecules such as proteins is energetically costly and it can therefore be hypothesized that these molecules are not just “leaking” into the urine after having served physiological functions in the body, but could play a role in chemical signalling in these species. Evidence of positive selection on WFDC12 during primate evolution suggests that this gene may be involved in sexual selection^57^. *WFDC12* transcripts are expressed in the human prostate as well as the skin, lungs and oesophagus^58^. Proteomics expression data (http://pax-db.org/protein/1855755/WFDC12) for human WFDC12 confirms that the protein has been detected in very few tissues (skin, whole organism aggregated data). In skin the protein expression level is at approximately 80 ppm, compared to the urine abundance that exceeds 10^5^ ppm. The exceptionally high level of seasonal output of this protein is remarkable. At present, it is not possible to establish the tissue of origin of the mouse lemur protein found in urine, nor is the tissue distribution of protein expression yet known, although it is tempting to suggest it may be a product of the lemur prostate gland that is activated in the reproductive season. However, the expression of a cDNA encoding sequence in female mouse lemur kidney (see previously) might be suggestive of other tissues of origin. The strong expression in voided urine is consistent with a specialised role rather than being part of the secretion of an active prostate gland; no other prostate-derived proteins are expressed at similar levels in urine (Fig. 1). Mouse lemurs display a behaviour known as ‘urine washing’, in which voided urine is deliberately deposited on hands, feet and subsequently on the substrate. Moreover, urine contains information about the dominance status of males^40^. It is possible that this protein is part of the scent of a dominant, sexually active male, but such a notion would require further exploration. From the analyses we have conducted thus far, there is no evidence for expression of MUPs in mouse lemur urine. However, it is unlikely that the role of the mouse lemur WFDC12 protein is volatile ligand binding, as it lacks the well-formed, beta barrel enclosed calyx that is a feature of MUP-like proteins. Thus, any role of the urinary WFDC12 protein is unlikely to be mediated by association with low molecular weight ligands.

## Additional Information

**How to cite this article:** Unsworth, J. *et al*. Characterisation of urinary WFDC12 in small nocturnal basal primates, mouse lemurs (*Microcebus spp.*). *Sci. Rep.* **7**, 42940; doi: 10.1038/srep42940 (2017).

**Publisher's note:** Springer Nature remains neutral with regard to jurisdictional claims in published maps and institutional affiliations.

## Supplementary Material

Supplementary Figures

Supplementary Table S1
