# Supplementary material for: Characterisation of urinary WFDC12 in small nocturnal basal primates, mouse lemurs (Microcebus spp.)
Source: Sci Rep. 2017 Feb 22;7:42940. doi: 10.1038/srep42940 (PMC5320513; doi:10.1038/srep42940)
Supplement: Supplementary Figures [file srep42940-s1.pdf]

Supplementary Figures

Characterisation of urinary WFDC12 in small nocturnal basal primates, mouse lemurs (*Microcebus spp.*)

Jennifer Unsworth, Grace Loxley, Amanda Davidson, Jane L Hurst, Guadalupe Gömez-Baena, Nicholas I Mundy, Robert J Beynon, Elke Zimmermann and Ute Radespiel

Supplementary Figure S1

Evidence for 'long' and 'short' forms of WFDC12 in *mouse lemur* male urine

Supplementary Figure S2

Evidence for 'long form' of WFDC12 in *Lemur catta (*ring-tailed lemur*)* urine (PEAKS DB & SPIDER)

Supplementary Figure S3

Evidence for an alternative splice form of WFDC12 in L catta.

Supplementary Figure S1
Evidence for 'long' and 'short' forms of WFDC12 in *mouse lemur* male urine (PEAKS DB)

a) Evidence for 'long' and 'short' forms of WFDC12 in *mouse lemur* male urine *Urine from mouse lemur males was digested with trypsin and analysed by LC-MS/MS. The peptides were matched to the 'long form' WFDC12 sequence using PEAKS software, and matched peptides were highlighted against the sequence using the PEAKSDB tool of the software. The map is intended to be illustrative, to indicate the extent (horizontal) and frequency of observations (vertical). Blue bars are database matches, grey bars are* de novo *sequence tags*

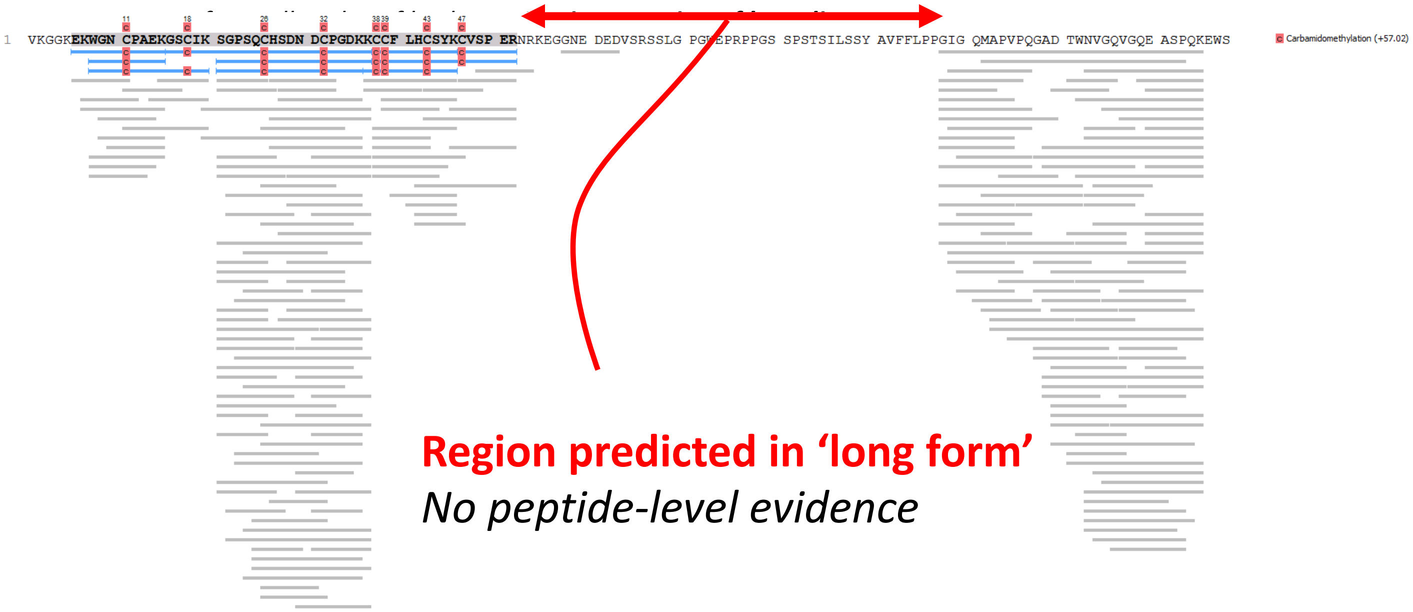

b) Evidence for 'short form' of WFDC12 in mouse lemur male urine (PEAKS DB)
Urine from mouse lemur males was digested with trypsin and analysed by LC-MS/MS. The peptides were matched to the 'short form' WFDC12 sequence using PEAKS software, and matched peptides were highlighted against the sequence using the PEAKSDB tool of the software. The map is intended to be illustrative, to indicate the extent (horizontal) and frequency of observations (vertical).

*Data are from all males of both species, **irrespective of breeding status or season***

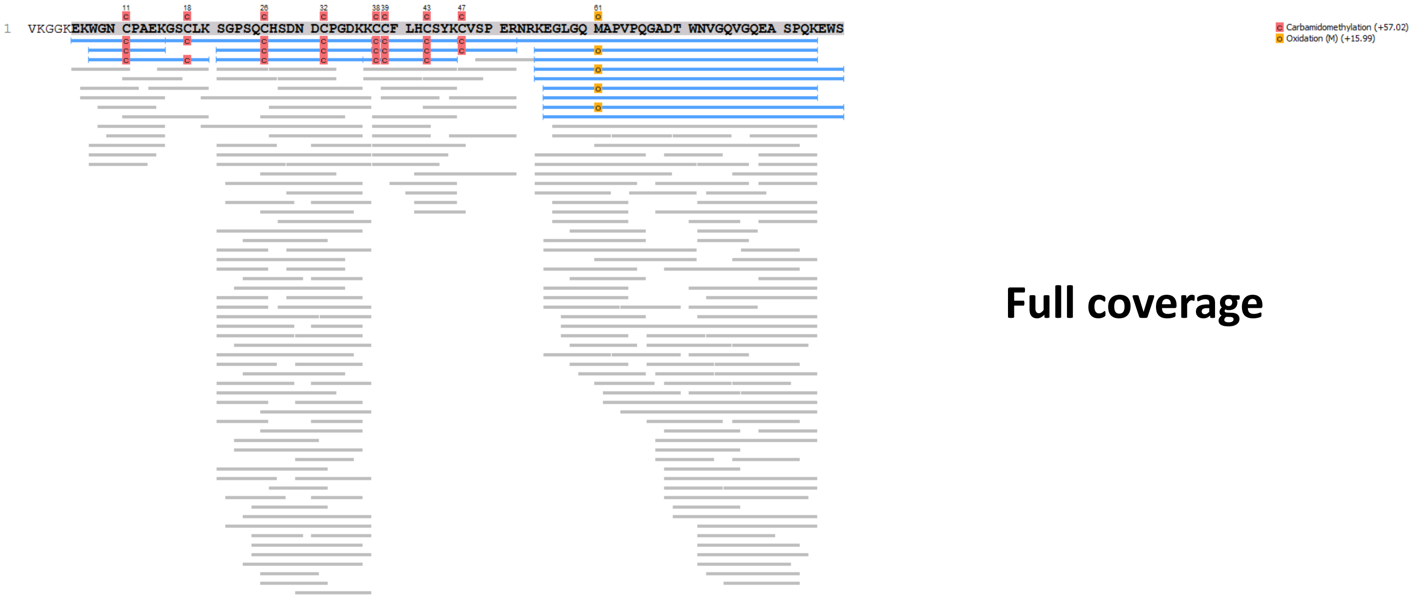

c) Evidence for 'long form' of WFDC12 in *M murinus* female urine (PEAKS DB)

Urine from mouse lemur females was digested with trypsin and analysed by LC-MS/MS. The peptides were matched to the 'long form' WFDC12 sequence using PEAKS software, and matched peptides were highlighted against the sequence using the PEAKSDB tool of the software. The map is intended to be illustrative, to indicate the extent (horizontal) and frequency of observations (vertical).

Data are from all females. The low degree of coverage, compared to males, reflects the substantially lower level of protein expression.

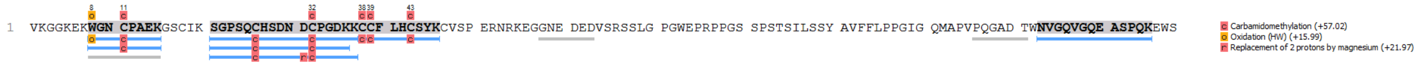

d) Evidence for 'short form' of WFDC12 in *M murinus* female urine (PEAKS DB)

Urine from mouse lemur females was digested with trypsin and analysed by LC-MS/MS. The peptides were matched to the 'short form' WFDC12 sequence using PEAKS software, and matched peptides were highlighted against the sequence using the PEAKSDB tool of the software. The map is intended to be illustrative, to indicate the extent (horizontal) and frequency of observations (vertical).

Data are from all females. The low degree of coverage, compared to males, reflects the substantially lower level of protein expression. The N-terminal matches are common to both short form and long form searches.

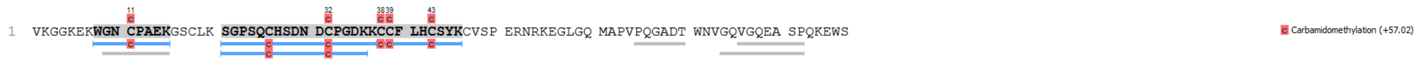

e) Evidence for 'long form' of WFDC12 in male mouse lemur urine (SPIDER)

Urine from mouse lemur males was digested with trypsin and analysed by LC-MS/MS. The peptides were matched to the 'long form' WFDC12 sequence using PEAKS software, and matched peptides were highlighted against the sequence using the SPIDER tool of the software, which performs exhaustive searches for MS/MS tag sequences. . The map is intended to be illustrative, to indicate the extent (horizontal) and frequency of observations (vertical).

Data are from all males of both species. The low degree of coverage, compared to males, reflects the substantially lower level of protein expression.

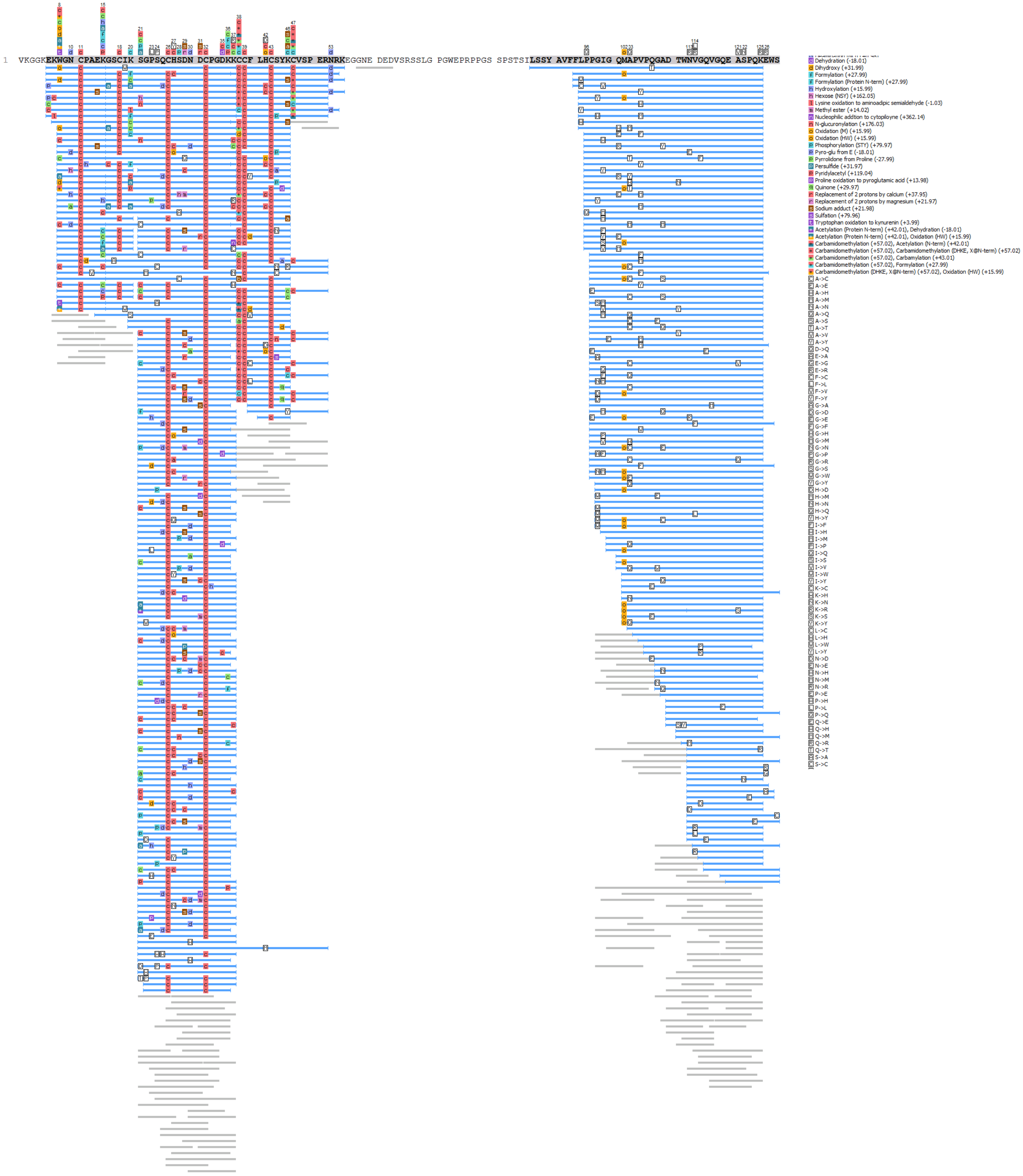

Evidence for 'short form' of WFDC12 in male mouse lemur in breeding season (SPIDER)

Urine from mouse lemur males (both species) was digested with trypsin and analysed by LC-MS/MS. The peptides were matched to the 'short form' WFDC12 sequence using PEAKS software, and matched peptides were highlighted against the sequence using the SPIDER tool of the software, which performs exhaustive searches for MS/MS tag sequences. . The map is intended to be illustrative, to indicate the extent (horizontal) and frequency of observations (vertical).

Data are from all breeding season males. The high degree of coverage reflects the substantially higher level of protein expression.

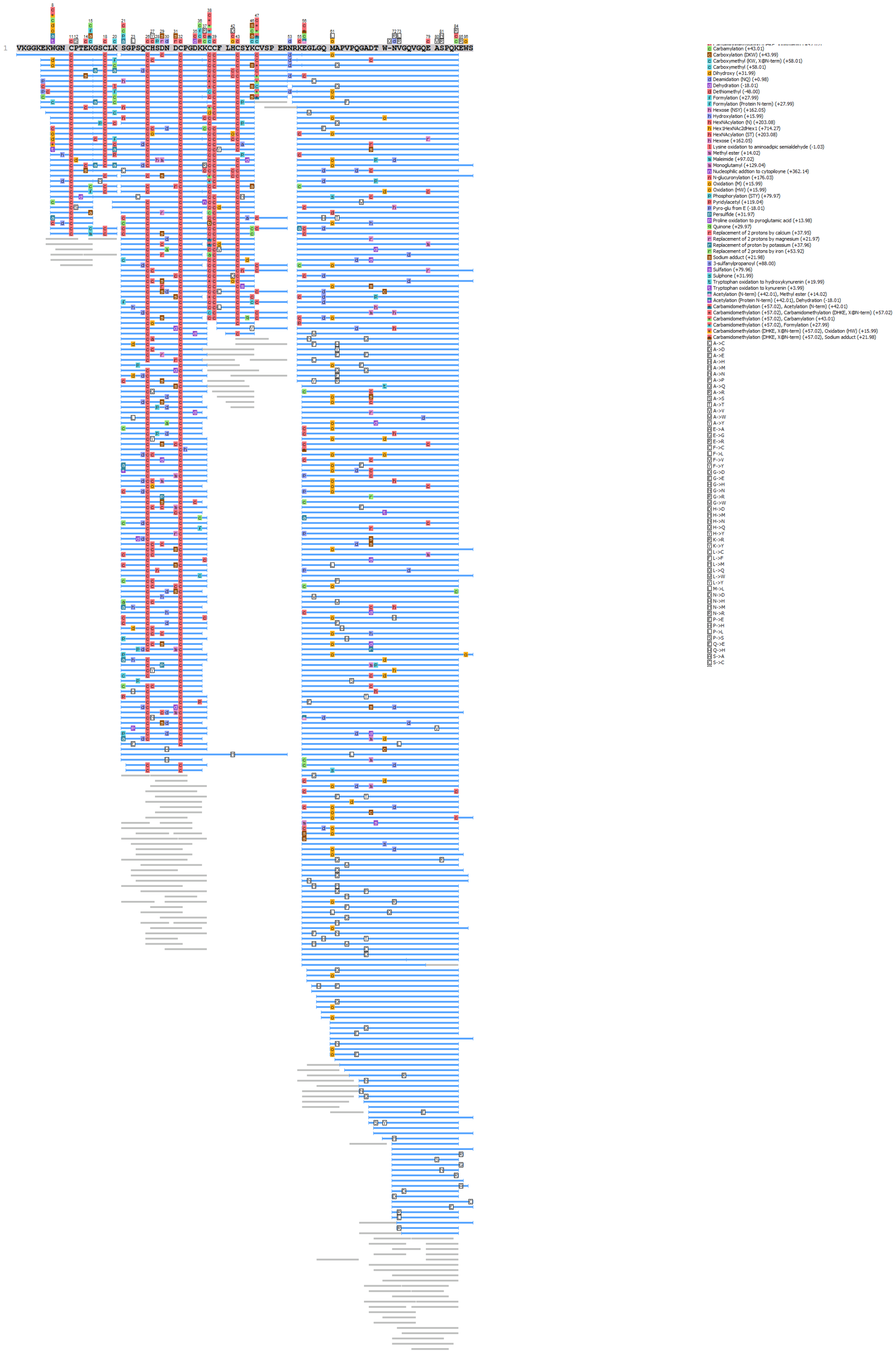

Evidence for ‘long form’ of WFDC12 in male mouse lemur urine out of breeding season (SPIDER)

Urine from mouse lemur males (both species) was digested with trypsin and analysed by LC-MS/MS. The peptides were matched to the ‘long form’ WFDC12 sequence using PEAKS software, and matched peptides were highlighted against the sequence using the SPIDER tool of the software, which performs exhaustive searches for MS/MS tag sequences. . The map is intended to be illustrative, to indicate the extent (horizontal) and frequency of observations (vertical).

Data are from all males out of breeding season. The low degree of coverage reflects the substantially lower level of protein expression.

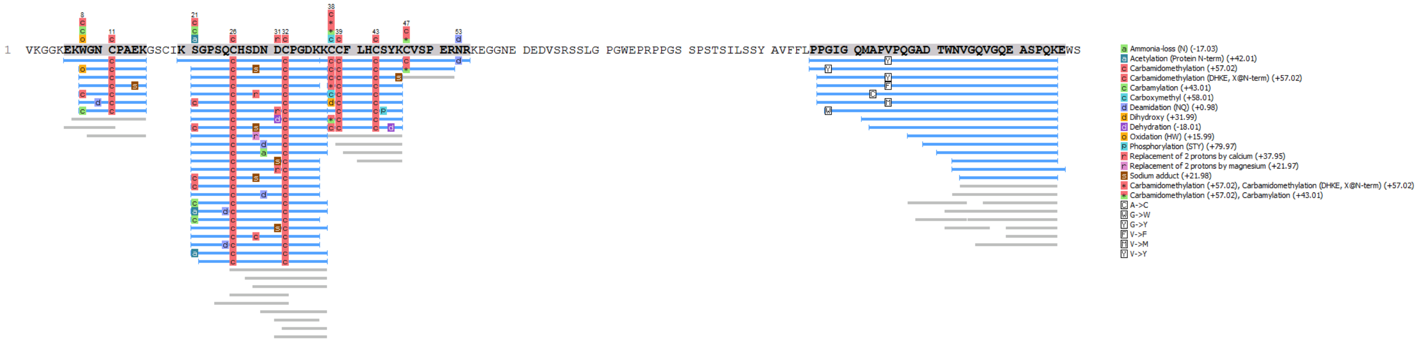

Evidence for ‘short form’ of WFDC12 in male mouse lemur urine out of breeding season (SPIDER)

Urine from mouse lemur males (both species) was digested with trypsin and analysed by LC-MS/MS. The peptides were matched to the ‘long form’ WFDC12 sequence using PEAKS software, and matched peptides were highlighted against the sequence using the SPIDER tool of the software, which performs exhaustive searches for MS/MS tag sequences. . The map is intended to be illustrative, to indicate the extent (horizontal) and frequency of observations (vertical).

Data are from all males out of breeding season. The low degree of coverage reflects the substantially lower level of protein expression.

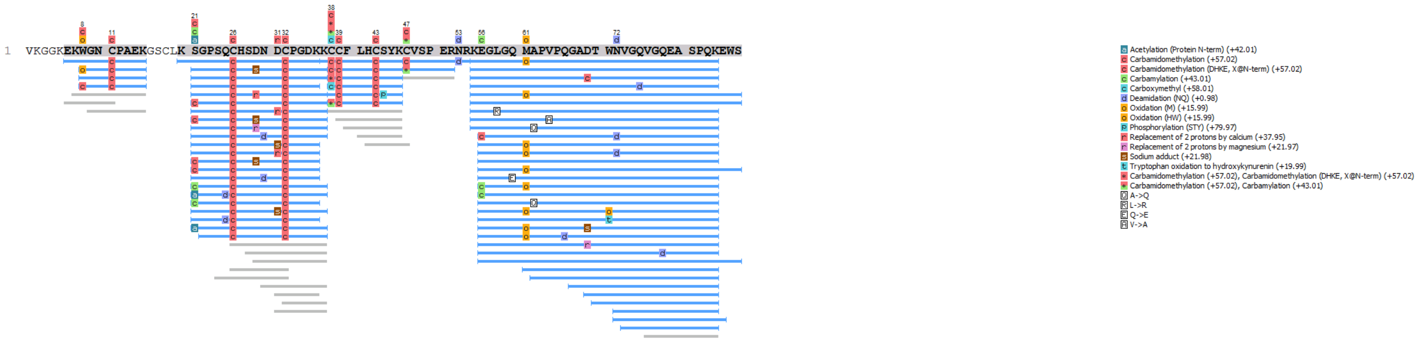

Evidence for ‘long form’ of WFDC12 in female mouse lemur urine (SPIDER)

Urine from mouse lemur females (M murinus only) was digested with trypsin and analysed by LC-MS/MS. The peptides were matched to the ‘long form’ WFDC12 sequence using PEAKS software, and matched peptides were highlighted against the sequence using the SPIDER tool of the software, which performs exhaustive searches for MS/MS tag sequences. . The map is intended to be illustrative, to indicate the extent (horizontal) and frequency of observations (vertical).

Data are from all males out of breeding season. The low degree of coverage reflects the substantially lower level of protein expression.

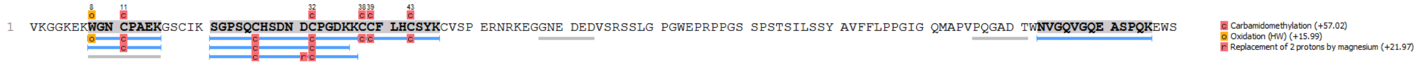

Evidence for ‘short form’ of WFDC12 in female mouse lemur urine (SPIDER)

Urine from mouse lemur females (M murinus only) was digested with trypsin and analysed by LC-MS/MS. The peptides were matched to the ‘short form’ WFDC12 sequence using PEAKS software, and matched peptides were highlighted against the sequence using the SPIDER tool of the software, which performs exhaustive searches for MS/MS tag sequences. . The map is intended to be illustrative, to indicate the extent (horizontal) and frequency of observations (vertical).

Data are from all females. The low degree of coverage reflects the substantially lower level of protein expression.

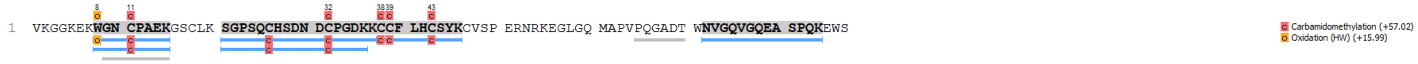

Supplementary Figure S2

Evidence for 'long form' of WFDC12 in *Lemur catta* (ring-tailed lemur) urine (PEAKS DB & SPIDER)

Urine from ring-tailed lemurs was digested with trypsin and analysed by LC-MS/MS. The peptides were matched to the 'long form' WFDC12 sequence using PEAKS software, and matched peptides were highlighted against the sequence using the PEAKSDB and SRIDER tool of the software. The map is intended to be illustrative, to indicate the extent (horizontal) and frequency of observations (vertical). Blue bars are database matches, grey bars are de novo *sequence tags*

[PEAKSDB]

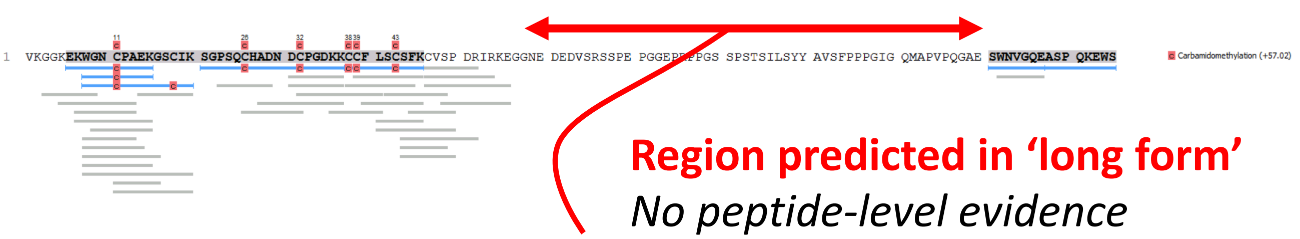

[SPIDER]

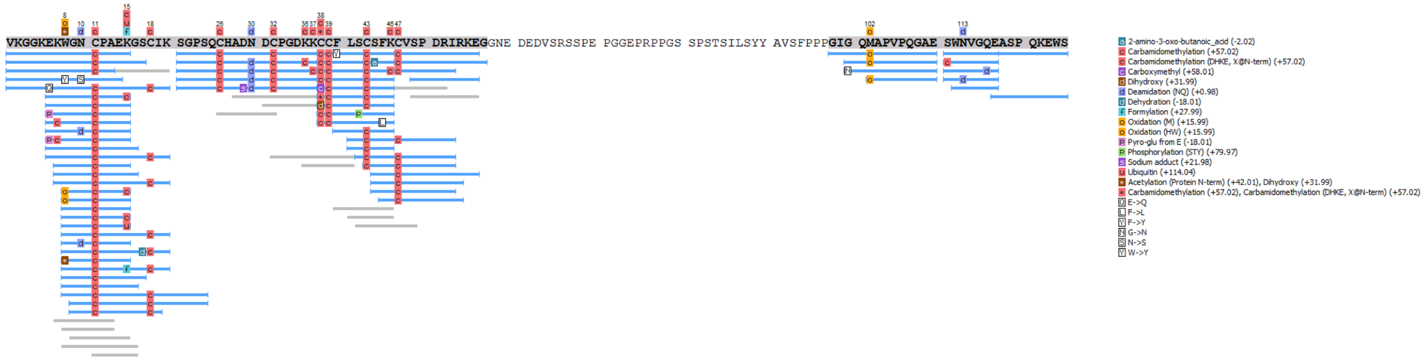

Evidence for 'short form' of WFDC12 in *Lemur catta* (ring-tailed lemur) urine (PEAKS DB & SPIDER)

[PEAKSDB]

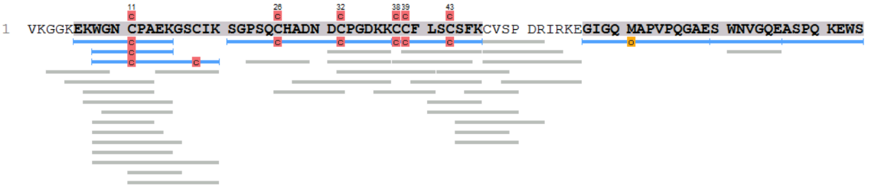

[SPIDER]

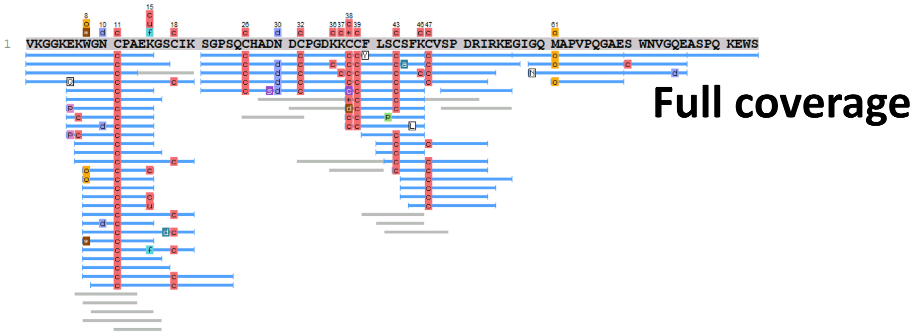

Supplementary Figure S3

Evidence for an alternative splice form of WFDC12 in *L catta.*
Based on the predicted sequence of *L catta*, together with MS/MS analysis of *L catta* urine (previous page), there is considerable evidence that the 'short form' also is the form expressed in *L catta* urine. There is clear potential for a splice variant (as in *M murinus*, see Figure 5, main text).

a)

```
exon 1  ATGAGGTCCTACAGCTTCTGGTTCCTTACAGCGTTCCTGGTTTTTGCGACCCTGGCGCTC
        GGGGAAGCAGTCAAAGGGGGTGAATGGATATGGTGGACCTGGGTGGGGCTGGGCTAGGGA
        GAGGCCCTGAGGGGCCGTCTGGGGCTGTAGGCAGTGCTGGA-CCCTCACCTCAACCCGTG
        GCTTCCTCCACCAGGTAAAGAGAAGTGGGGAAATTGCCCAGCCGAGAAAGGAAGCTGCAT
exon 2  CAAGTCCGGTCCGTCCCAGTGTCATGCCGATAATGACTGTCCTGGGGACAAGAAATGCTG
        CTTCCTGAGTTGTAGCTTCAAATGTGTGAGTCCTGACAGGATCAGAAAGGAAGGTAAGGA
        GCCCCTGCATACCAGGCCTGGTGCTCTCACTTCCCCTGCCTCTGTCTGGCCCATGAAAGC
        ACGGAGGAATTAGTGCTTTTAGGCGGTGTGGGGAGGGATGGTTAGAGTTGGCAGGGCCCT
        AAGAGATCAACAAGTCTGAACATCTCCATTGCACAAAGGGGGAAATGAGGATGAAGATGT
        GTCAAGATCATCCCCAGAGCCAGGAGGGGAGCCCAGGCCTCCAGGTTCGTCCCCTTCCAC
        AAGTATCCTCAGTTATTATGCTGTGTCCTTTCCACCTCCAGGTATCGGTCAAATGGCACC
exon 3  TGTTCCTCAGGGTGCTGAATCTTGGAAT---------GTGGGACAAGAAGCGAGCCCCCA
        GAAGGAATGGTCCTGA
```

b)

```
                   exon1                      exon2
Lemur catta        [MRSYSFWFLTAFLVFATLALGEA]VKGGKEKWGNCPAEKGSCIKSGPSQCHADNDCPGDKKCCFLSCSFKCVSPDRIRKE
M murinus pred     [MKTLCFLVLTAFLVIATLTPGEA]VKGGKEKWGNCPAEKGSCIKSGPSQCHSDNDCPGDKKCCFLHCSYKCVSPERNRKE
M murinus obs      <----Signal peptide---->  VKGGKEKWGNCPAEKGSCJKSGPSQCHSDNDCPGDKKCCFJHCSYKCVSPERNRKE

                   exon3
Lemur catta        -GNEDEDVSRSSPEPGGEPRPPGSSPSTSILSYYAVSFPPPGIGQMAPVPQGAESWN---VGQEASPQKEWS
M murinus pred     GGNEDEDVSRSSLGPGWEPRPPGSSPSTSILSSYAVFFLPPGIGQMAPVPQGADTWNVGQVGQEASPQKEWS
M murinus obs                                               GJGQMAPVPQGADTWNVGQVGQEASPQKEWS
                                                            |
                                                 Alternate splice
                                                 junction, exon 3
```
